# Self-assembly of fractal liquid crystal colloids

**DOI:** 10.1038/s41467-018-08210-w

**Published:** 2019-01-14

**Authors:** Nikita V. Solodkov, Jung-uk Shim, J. Cliff Jones

**Affiliations:** School of Physics and Astronomy, University of Leeds, Leeds, LS2 9JT UK

## Abstract

Nematic liquid crystals are anisotropic fluids that self-assemble into vector fields, which are governed by geometrical and topological laws. Consequently, particulate or droplet inclusions self-assemble in nematic domains through a balance of topological defects. Here, we use double emulsions of water droplets inside radial nematic liquid crystal droplets to form various structures, ranging from linear chains to three-dimensional fractal structures. The system is modeled as a formation of satellite droplets, distributed around a larger, central core droplet and we extend the problem to explain the formation of fractal structures. We show that a distribution of droplet sizes plays a key role in determining the symmetry properties of the resulting geometric structures. The results are relevant to a variety of inclusions, ranging from colloids suspensions to multi-emulsion systems. Such systems have potential applications for novel switchable photonic structures as well as providing wider insights into the packing of self-assembled structures.

## Abstract (web summary)

Topological defects in liquid crystals can drive the assembly of colloidal inclusions. Here the authors place water droplets of different sizes in a larger nematic liquid crystal drop and observe the formation and self-assembly of fractal colloidal structures.

## Introduction

In a world governed by symmetry and invariance principles, complex mathematical concepts often realize themselves in seemingly simple physical systems^1^. Topology is a study of geometrical properties that are preserved by continuous deformations, which is mostly associated with pure mathematics^2^. However, it provides coherence to many physical phenomena that are not always abstract, including cosmology^3^, topological insulators^4^ and flow fields in fluid mechanics^5^.

Liquid crystals are anisotropic fluids, in which the rigid and anisotropic constituent molecules have a strong tendency to form mesophases with long-range orientational order, described by the order parameter *S*. In the simplest and most widely studied liquid crystal phase, the nematic, the molecules are free to move around each other and lack any long range positional order. The molecular symmetry axes within an ensemble share a common pointing direction, described by a unit pseudo-vector **n**, called the director. Nematic liquid crystals are described by cylindrical symmetry and are uniaxial, which makes **n** physically indistinguishable from −**n**^6^. Many of the physical properties, such as electric permittivities and elastic constants^7^, are anisotropic and related to **n**. Much like normal mathematical vector fields, director fields are governed by geometry, boundary conditions and topological properties of the domain containing it^8–11^. For example, a spherical nematic droplet with radial boundary conditions must contain a radial-like singularity^9,12^, known as a topological defect^13^ (cf. sink/source in fluid mechanics). This singularity can be replaced by any set of topologically equivalent structures, such as a defect loop or a combination of different defects^2,10–12^. To describe the interactions of these singularities, each one is assigned a property known as the topological charge. This is a measure of the number of times the director turns around a closed loop or surface of an isolated singularity. From a topological point of view, the sum of topological charges *q*_*i*_ is an invariant in a closed domain with fixed boundary conditions^2^. Therefore, a forced defect in a nematic director field will result in the creation of an accompanying defect of opposite topological charge^10–12^.

While defects are typically avoided in devices, their presence is essential in some systems, where they act as one of the primary device mechanisms. An early example of this is the Zenithal Bistable Display, in which a deep, surface relief grating with normal director boundary conditions is used to induce defects that stabilize a low tilt alignment state, or that allow a continuous high tilt state^14–16^. The defects are electrically induced at the point of inflexion on the vertical edges of a grating surface and then separated using the polar response that arises from strong elastic deformations due to the inherent flexoelectricity of the nematic phase^17,18^.

More recently, there has been an explosion of interest in micro-suspensions within nematic liquid crystals, wherein the intrusions self-assemble due to the creation of defects in the nematic director fields^19–25^. This process was first observed by creating water droplets with radial boundary conditions inside larger nematic droplets, which created linear chains of inclusions^19^. In such systems, topologically forced hyperbolic defects stabilize the suspensions of particles by forming topological dipoles with the radial inclusions, which line up in a similar fashion to a linear array of electric dipoles^11,20^. Similar systems have been observed by adding micro-particles with pre-determined boundary conditions and locally melting the director field with laser tweezers to control the formation of structures through manual rearrangement of inclusions^26^. Other studies, such as^27^, aim to create unique defect combinations by adding holes to particles, which changes their topological properties.

In this work, we use double emulsion droplets generated in a microfluidics device and controlled agitation to create multiple water droplets with radial boundary conditions inside larger radial nematic droplets. We find that the size differences between the water droplets play a key role in the spontaneous formation of complex three-dimensional (3D) structures, ranging from linear chains to fractal structures. To explain our observations, we use numerical analysis to relate the basic formation of colloidal structures in radial nematic droplets to the solutions of the Thomson problem^28,29^ and extend the analogy to the formation of fractal structures. In contrast to a recent study by Hashemi et al.^30^ that studies the behavior of nematic defects along predetermined fractal shapes, we observe spontaneous formation of fractal shapes due to the topological and elastic properties of nematic liquid crystals.

## Results

### Critical distortion

Consider the domain of a nematic droplet with radial (normal to the surface) boundary conditions. From topological principles, a director field discontinuity with a charge of +1 is formed inside it and centralized to minimize the free energy of the system. Adding a smaller inclusion (such as a particle coated with a homeotropic surfactant, or a second water droplet) with normal boundary conditions does not create any additional distortion to the radial director field. Instead, the inclusion minimizes the free energy by creating a virtual, highly splayed defect at the center of the particle and moving the inclusion to the center of the system. In this work, we will refer to the first inclusion (water droplet) as the core and additional +1 radial inclusions as satellites, which must be accompanied by −1 hyperbolic defects to conserve the total topological charge of the system.

**Fig. 1:**
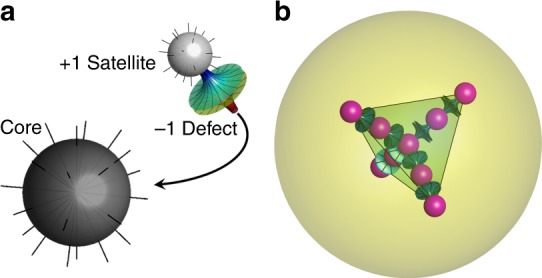
Inclusion self-assembly process. **a** Illustration of the satellite to core attachment process. **b** Diagram showing a topologically stabilized structure with tetrahedral symmetry: water droplets are shown by magenta balls, hyperbolic defects are shown in cyan and the nematic region is shown in yellow

For illustration purposes we assume that the core always remains at the centroid of the nematic domain, which is true for the cases of 3D symmetric structures. Once a satellite enters the director field, elastic forces drag it towards the point of highest splay, where it enters the core’s primary orbit. All other satellites entering this primary orbit are attracted to the core but repel each other. This suggests that there exists a maximal capacity of satellites in the primary orbit $N_{\mathrm{1}}^{\mathrm{c}}$. By symmetry, the second satellite must attach itself on the opposite side of the core to the first, to minimize elastic distortion. A third satellite then has a choice between readjusting the positions of the first two satellites and attaching itself to the core or to one of the two existing satellites co-linearly. The relative sizes of the droplets that determine the resulting structure. For droplets of similar size, the third and fourth droplets will become arranged at the tetrahedral angles to the core, and additional droplets beyond the fourth attach as satellites to the higher orbitals, four per orbital, to create a characteristic tetrahedral structure. Figure 1 shows the satellite attachment process and a tetrahedral structure (first 3D shape) with linear chains extending radially outward from the core that results from near equivalent sized internal droplets.

**Fig. 2:**
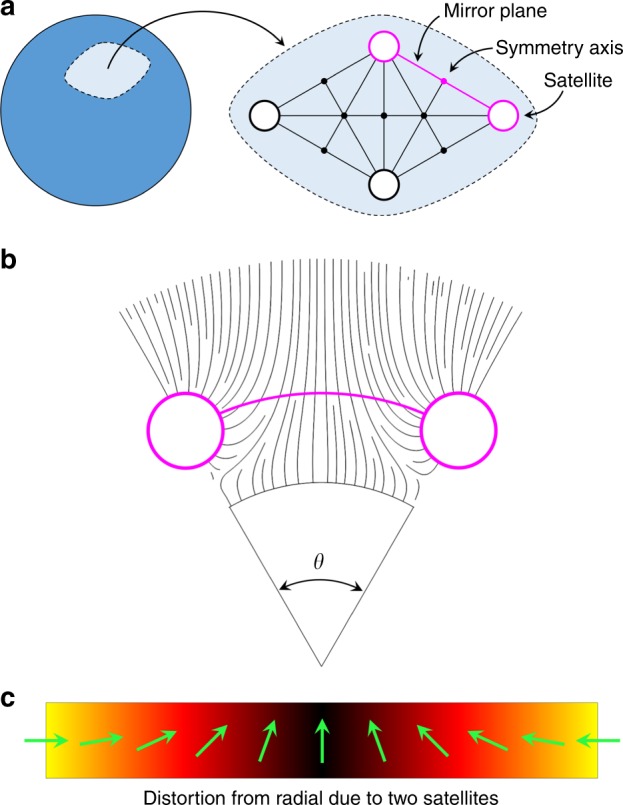
Satellite distortion network. **a** Sub-domain of the spherical surface corresponding to the primary satellite orbit. Large unfilled circles represent satellites, small filled circles represent symmetry axes of rotation with radial director profiles and solid lines represent unfolded mirror planes. The plane of highest disturbance (repeated in the structure) is highlighted in magenta. **b** A slice of the magenta plane from **a**, showing the director streamlines due to two satellites separated by a polar angle of *θ*. **c** Distortion of the director field in the radial reference frame as a function of the polar angle between two neighboring satellites along their orbital path, magenta arc from **b**

To determine the maximal orbit capacity, we first need to study the solution sets for distributions of repulsive points around the boundary of a circle in 2D and on the surface a sphere in 3D. In 2D systems, primary orbit satellites distribute themselves along the vertices of regular polygons. Similarly, in 3D the structures follow the solutions to the Thomson problem (originally used to describe the electronic structure of atoms for the superseded “plum-pudding” model), which include some regular polyhedrons. Two satellites can continue to move closer together until the director reaches a critical distortion (this is equivalent to adding more satellites to the orbit). By symmetry of the director field, primary orbit satellites share a network of mirror planes and symmetry axes of rotation (see Fig. 2). We can see that the highest amount of distortion in the director field occurs in the plane containing the core and two nearest neighbor satellites. As the two satellites in the same orbit approach each other, the critical separation point will be reached first in this plane, thereby reducing a 3D problem to 2D.

Each satellite of unitary radius is pulled towards the core of radius *r* to minimize the system’s elastic free energy and stabilized at a center-to-center separation of (*r* + 1)*h*, where *h* is the separation factor. We find that *h* = 1.24 for the nematic liquid crystal E7 at room temperature, which closely follows the values determined numerically^31^ and experimentally^19,32^ for similar materials when in dipolar chains. Due to the symmetry of the system, we consider two elastic constants: *K*_1_, which opposes the divergence of the director field, and *K*_3_, which opposes the bending of director streamlines. In our system, *K*_3_ is responsible for pushing orbit-equilibrated satellites away from each other. On the other hand, *K*_1_ restores the director field to the radial configuration, which allows satellites to be closer to each other. This implies that there exists a critical angular separation of neighboring satellites *θ*_c_, beyond which no two satellites can come closer without a great cost to the elastic free energy of the system.

To determine the critical separation of satellites we study the natural distortion of the director field that a lone satellite creates. Since we seek the critical value, only the distortion along the orbit needs to be examined, where the repulsion between adjacent satellites is greatest. At the boundary of the satellite, the distortion is maximal and decays away as a function of the polar angle at a rate that depends on the ratio of splay *K*_1_ and bend *K*_3_ elastic constants. Introducing a second satellite to the same orbit is equivalent to creating a mirror line half way between them, where the director is fully straightened to the radial configuration. *θ*_c_ is reached at the point where the natural distortion angle from the radial configuration of the lone satellite reaches a critical value of $\xi _{\mathrm{c}} = {\mathrm{tan}}^{ - 1}\left( {K_1{\mathrm{/}}K_3} \right)$. This means that two satellites can be brought closer together up to the point where their push (i.e. *K*_3_) dominant regions touch.

As the core to satellite size ratio *r* increases, we expect the polar influence of satellites to become less significant and *θ*_c_ to decrease with it, allowing a greater number of satellites to enter the primary orbit. At low *r*, satellites are closer to the center of the domain and their natural director profiles closely match each other, which decreases *θ*_c_. On the other hand, the natural director field of the domain becomes less divergent with increasing orbit size and streamlines become more parallel. By comparing the director field to the electrostatic field lines between two charges, we make an ansatz of the following form

1 $$\theta _{\mathrm{c}} = 2\theta _0 - 2\alpha \frac{{\xi _{\mathrm{c}}}}{{\sqrt r }}{\mathrm{ln}}\left( {\frac{{\xi _{\mathrm{c}}}}{{\xi _0}}} \right),$$

where *α* is constant for a given domain size, $\theta _0 = 2\,{\mathrm{csc}}^{ - {\mathrm{1}}}\left( {2h(r + 1)} \right)$ and *ξ*_0_ = (*π* − *θ*_0_)/2 are the polar and the distortion angles at the boundary of the satellite, respectively. From this, we can expect that liquid crystals with high *K*_1_/*K*_3_ ratios will be able to support more satellites than the ones with low *K*_1_/*K*_3_ ratios.

### Primary orbit satellite packing

**Fig. 3:**
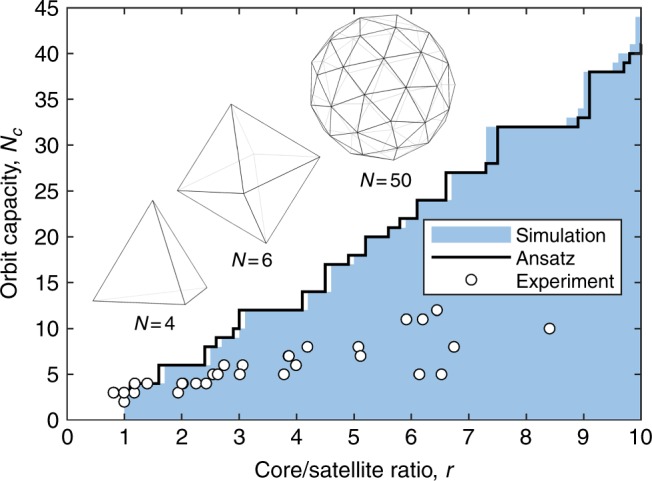
Primary orbit capacity and packing. Orbit capacity *N* as a function of the core to satellite size ratio *r* illustrating numerical space of possible values (blue shading), estimated maximal orbit capacity from the ansatz (black line), a representative selection of experimental results achieved with water in E7 in water double emulsions (circles). Shapes corresponding to *N* = 4, *N* = 6, and *N* = 50 are shown above the curves

The maximal number of satellites in the primary orbit $N_{\mathrm{1}}^{\mathrm{c}}$ can be calculated numerically by comparing *θ*_c_ with the angles generated by the closest neighboring vertices of the Thomson problem solutions. As *r* tends to infinity, exact solutions of $N_{\mathrm{1}}^{\mathrm{c}}$ can be found using the Fejes inequality^28^, which also holds for $N_{\mathrm{1}}^{\mathrm{c}}$ equal to 2, 3, 4, 6, and 12. The value of $N_{\mathrm{1}}^{\mathrm{c}}$ serves as the maximal achievable number of primary satellites for a given core to satellite ratio of the system. We find that our ansatz is closely matched by the numerical solution with *α* = (1.12 ± 0.01) and therefore, can be used as quicker estimation method for $N_{\mathrm{1}}^{\mathrm{c}}$ as a function of *r*. This can be seen in Fig. 3, which shows the allowed solutions for the primary orbit satellites alongside a representative selection of experimental results. The Fejes inequality^28^ for *N* points on a unit sphere states that

2 $$2\,{\mathrm{sin}}\left( {\frac{\theta }{2}} \right) \le \left( {4 - {\mathrm{csc}}^2\left( {\frac{{\pi N}}{{6(N - 2)}}} \right)} \right)^{\frac{1}{2}},$$

which becomes exact for *N* = 3, 4, 6, 12 and *N* → ∞. Due to the dependence of *θ*_c_ on *r* and the associated Thomson problem solution for *N*, we find that the relationship between $N_{\mathrm{1}}^{\mathrm{c}}$ and *r* is weakly non-linear for small values of *r* and increases in a step-function-like fashion. The numerical results indicate that for systems in which the satellites are identical in size to their cores, triangular configurations are expected and we may expect tetrahedral structures to form once *r* reaches 1.1. In practice, there always exists a small size distribution of water inclusions, which creates enough variation in *r* to allow tetrahedral structures.

Experimental observations of double emulsions formed using microfluidics indicate that satellite droplets self-assemble into tetrahedral configurations almost exclusively and lower order configurations are possible but rarely observed. This is a direct consequence of the fact that the nematic droplets prefer to have spherical symmetry and for the contained structures to match it. This introduces an additional balance between spherical and polyhedral symmetries. Digonal and triangular configurations are two-dimensional and lack 3D symmetry balance. On the other hand, the tetrahedral configuration is the lowest order structure with 3D symmetry balance and is therefore preferred, as observed in double emulsions. To achieve configurations with two and three primary satellites in 3D nematic droplets, a reduction in symmetry must be introduced. The digonal configuration can be achieved by forcing the nematic droplets to have an ellipsoidal shape during a slow self-assembly process. Following their assembly, such structures remained stable throughout the observation period (over a week), due to the stabilizing effects of the topologically imposed hyperbolic defects separating the inclusions at the centers of the nematic droplets. When the samples were heated above the nematic to isotropic phase transition temperature, this stabilization disappeared (due to the lack of a director field) resulting in collapse of the structures into a single, larger core.

**Fig. 4:**
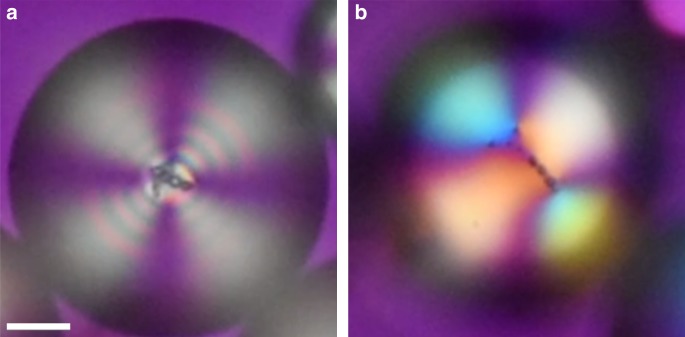
Switching between primary satellite configurations. Polarized microscopy images of **a** a nematic droplet with a tetrahedral structure in the center and **b** the same nematic droplet after external agitation on the brink of the nematic to isotropic phase transition. Full waveplate is inserted at 45° to the crossed polarizers to confirm radial orientation of the director field. Scale bar: 10 μm

Once a structure is formed inside a nematic droplet, it can be switched into a different configuration by applying an external field. Here we illustrate this by switching a tetrahedral structure inside a 3D radial nematic droplet into a lower order state. To achieve this effect, the droplet was deformed rapidly by applying external pressure to the observed region of the glass containers (used for observation under the microscope). This resulted in an unfolding of the tetrahedral structure formed naturally by the inclusions, followed by an immediate reconstruction into the closest energy minimum. By increasing the agitation, the structure was deformed sufficiently to reconstruct itself into a 2D shape while still remaining in a spherical 3D nematic droplet. A comparison between the original and the reformed structures is shown in Fig. 4. The resulting structure consisted of a triangular segment with a single linear chain of satellites. This bares a clear resemblance to the structures seen in 2D nematic droplets from ref. ^19^. Heating the sample close to the nematic to isotropic phase transition temperature reduces the number of birefringence fringes and allows a clearer comparison with the 2D cases.

**Fig. 5:**
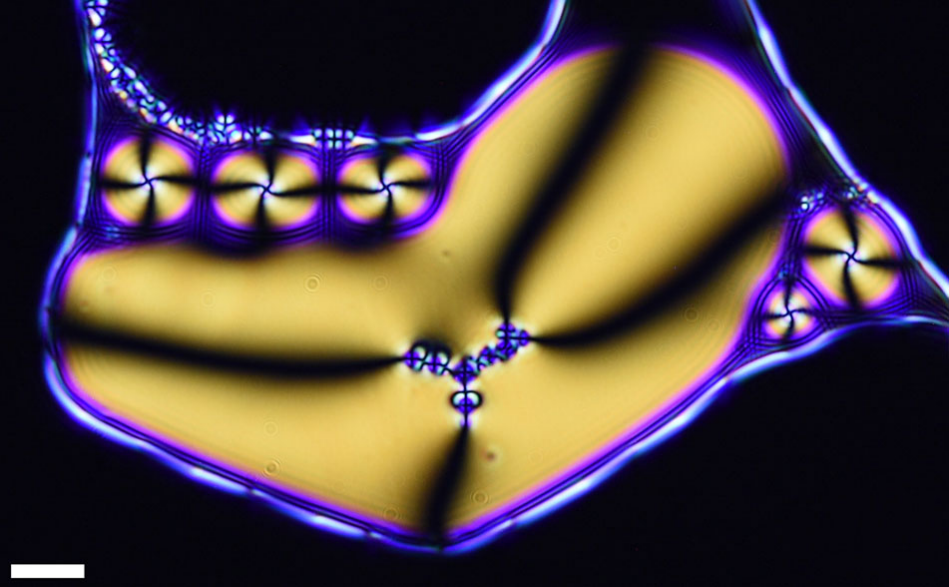
Triangular satellite self-assembly in 2D. Polarized microscopy image of a nematic droplet in the shape of a deformed disc in 2D space (third dimension close on the inclusion diameter scale) with normal boundary conditions. Inside the nematic domain, a 2D structure comprising of linear and triangular segments of inclusions with radial boundary conditions is formed, mimicking the shape of its container. Scale bar: 10 μm

Another way to switch the structures is by reducing the dimensionality of the samples. This can be done by reducing one of the coordinates to a length scale closely comparable to the inclusion diameter, for example, using a lateral force. Analogous to the 3D case, the satellites form structures following the vertices of regular polygons. When a droplet with a 3D structure is collapsed to a (relatively) flat disc, the geometrical shape is no longer supported by the dimensionality of the space and must collapse into a 2D configuration. Since $N_{\mathrm{1}}^{\mathrm{c}}(r \approx 1) = 3$, flattening a tetrahedral structure without an additional bias in a particular dimension results in a triangular structure. However, if a nematic droplet is deformed into a disc-like shape with a non-circular boundary, it results in a reorientation of the structure to mimic the geometrical asymmetry formed by the boundary. This is expected, as geometrical constraints extend their influence throughout the bulk of the nematic domain that they contain. To achieve this, a glass plate was freely suspended on the surface of a sample with radial nematic droplets, containing tetrahedral structures, in water. Evaporating the external water resulted in the aggregation and flattening of the nematic droplets and a collapse of 3D structures formed by the inclusions. As before, the resulting structures showed digonal, triangular symmetries and their combinations. An example of this is shown in Fig. 5, where we can see a combination these shapes, which form a structure that resembles the shape of the deformed disc-like container.

**Fig. 6:**
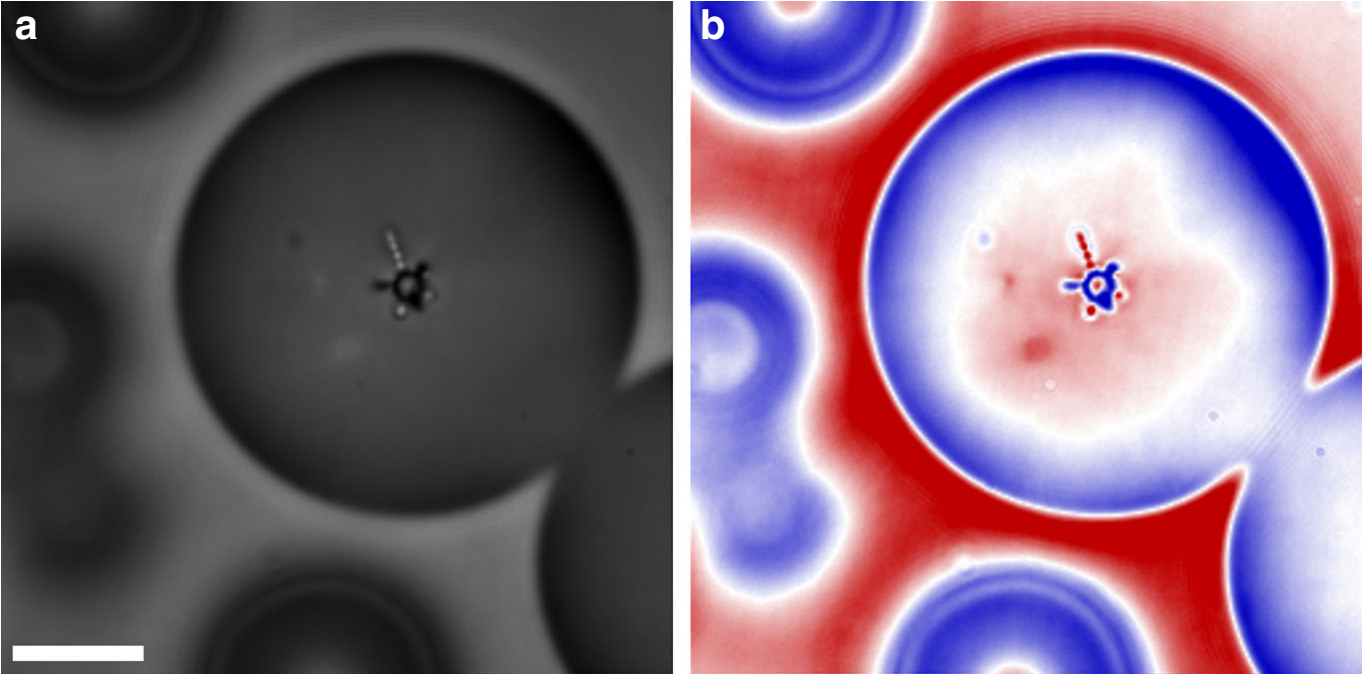
Octahedral satellite packing. Confocal microscopy image of a radial nematic droplet showing a structure with 6 primary orbit satellites formed by water droplets with normal boundary conditions **a** and the corresponding color enhanced image **b**. Scale bar: 10 μm

Increasing *r* further creates more docking sites in the primary orbit, allowing higher values of *N* to be achieved. However, most experimental observations are unlikely to reach the maximal orbit capacity limit for high *r*. Instead, each primary orbit satellite becomes a potential docking site for the nearby dock-seeking satellites, decreasing the statistical probability of subsequent primary orbit satellite attachments. An example of this can be seen in Fig. 6, which shows a confocal microscopy image of a radial nematic droplet with a structure comprised of 6 primary orbit satellites (forming the vertices of an octahedron) for *r* = 3.0 ± 0.5 (error from pixel size). The corresponding $N_1^{\mathrm{c}}$ for this core to satellite ratio from Fig. 3 is 10 ± 2, which suggests that the core had the potential to support up to 4 ± 2 additional satellites in its primary orbit.

### Fractals

Fractal structures are generated by self-similar patterns, consisting of rescaled copies of themselves. They are often seen in naturally occurring systems, ranging from snowflakes and seashells to the properties of the human heart^33^, diffusion limited aggregation^34^, Brownian motion^35^ and galaxy distributions^36^. To measure the self-similarity properties of fractals, we often use the (Hausdorff) fractal dimension, *d*, which describes the scale-independent change in detail of a fractal and its ability to fill space. For a given fractal pattern consisting of an initiator and a generator that produces *n* copies of its previous evolution, scaled down by a factor of 1/*r*, the fractal dimension is given by^37^

3 $$\mathop {\sum}\limits_{i = 1}^n {\kern 1pt} r_i^{ - d} = 1.$$

If the scaling factor *r* is the same for all evolution sites, then this equation reduces to *d* = log *n*/log *r*. For example, the Cantor set is constructed by repeatedly removing the middle third of a line segment at every step of the evolution. This results in the fractal dimension of log 2/log 3 ≈ 0.63, which does not have enough information to fill a 1D space. However, natural fractals often consist of finite number of evolution steps with irregular generators, which causes the fractal dimension to differ between evolution levels, as well as within them.

In cases such as the one from Fig. 6, the remaining space in the primary satellite orbit is not filled due to a small total number of inclusions. When there is a much larger number of inclusions, another self-assembly process can take over expanding the space of possibilities to the creation of fractal structures.

**Fig. 7:**
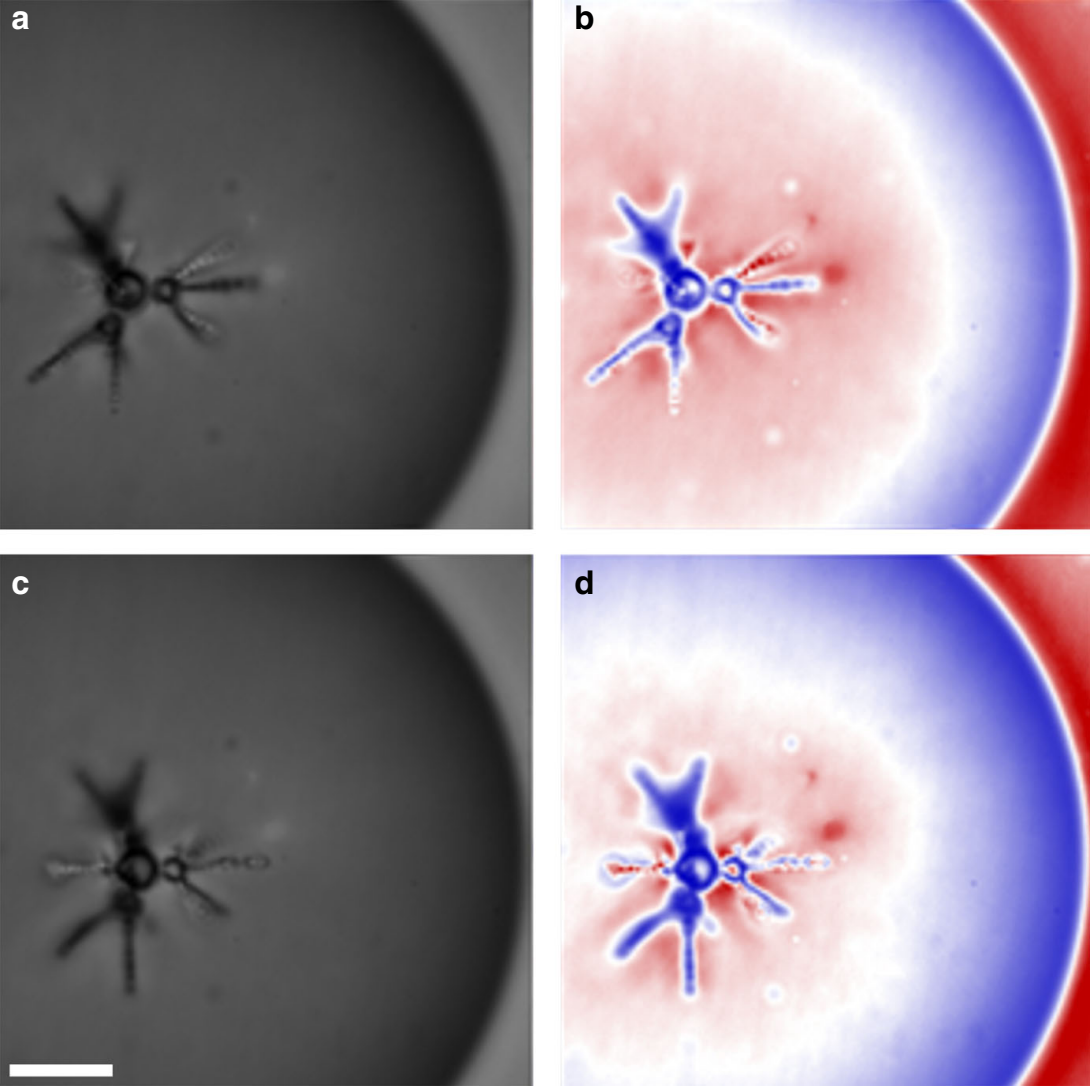
Fractal satellite packing. Confocal microscopy images showing a self-assembled fractal structure with 2 steps of evolution, formed by water inclusions with normal boundary condition inside a radial nematic droplet. Images **a**, **c** show different orientations of the same structure. Images **b**, **d** show the corresponding visually enhanced equivalents. Scale bar: 10 μm

As satellites self-assemble into chains that extend radially outwards from the core (see Fig. 1), they provide local distortion fields similar to that of the original core droplet. By symmetry, each droplet in a linear chain of satellites is separated by a series of warped planes that lie perpendicular to the pointing directions of each chain. Since the satellites have normal boundary conditions, each separator plane is equivalent to a 2D disc with radial boundary conditions. This means that a satellite situated between two neighboring separator planes can act as a secondary core, which acts as an additional docking space for much smaller secondary satellites. The self-assembly process of producing secondary satellites is very similar to that of the initial 3D problem, but now the structures follow the vertices of regular polyhedrons, centered at a secondary core (cf. Fig. 5). Figure 7 shows confocal microscopy images (taken from two different perspectives) of a fractal structure with 2 steps of evolution. The initial arrangement of the primary orbit satellites forms a tetrahedral base structure with *r* = 2.0 ± 0.4, which is extended at three of the four primary satellite chains into additional secondary structures with deformed triangular symmetry or *r* = 2.9 ± 0.6. Applying Eq. (3) before averaging gives *d* = 2.1 ± 0.4 for the first evolution level and *d* = 1.0 ± 0.2 range for the second evolution levels. With the aid of depth based color enhancement, we can clearly see that the structures are three-dimensional and follow the shapes described by our hypothesis.

**Fig. 8:**
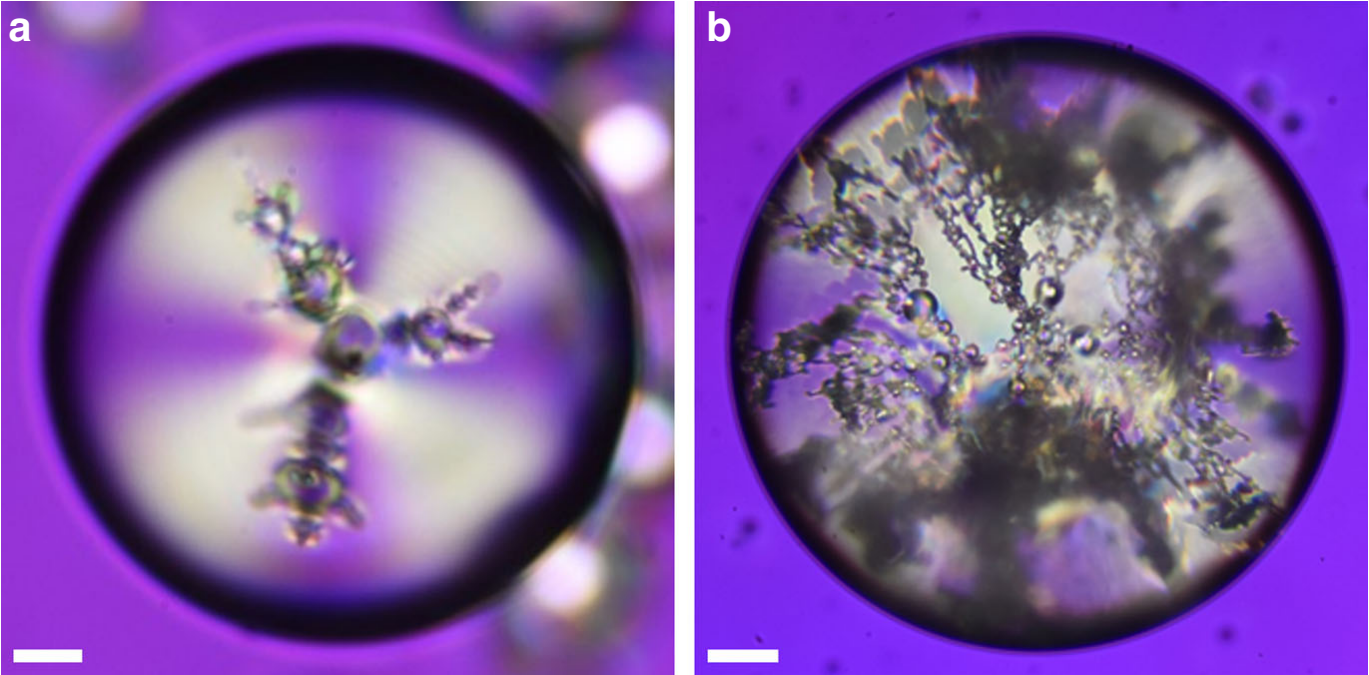
Large scale, complex fractal satellite packing. Polarizing optical microscopy photographs of **a** a nematic droplet with a fractal colloidal structure (tetrahedral frame with another arm behind the focal plane) and **b** a nematic droplet (450 μm radius) with an incoherent cascade of fractal structures formed by water inclusions with normal boundary conditions. A full waveplate is inserted at 45° to the crossed polarizers to see the 3D structure clearly. Retardation difference from the waveplate is screened by the high retardation due to the thickness of the nematic droplet. Scale bar **a:** 10 μm, scale bar **b**: 100 μm

We can extend this analogy further by allowing secondary satellites to act as tertiary cores for much smaller quaternary satellites, and so on. An example of this can be seen in Fig. 8a, which shows a polarizing optical microscopy image of a fractal structure progressing through several levels of evolving symmetry. The structure consists of a tetrahedral base of primary satellites (fourth primary chain hidden in the image due to the viewing angle and the location of the focal plane) and extends into a series of fractal structures with 3 or more steps of evolution. This suggests that if there exists a sufficiently large number of inclusions with a wide size distribution inside a radial nematic droplet, then a fractal structure will form around the core. Evaluating the fractal dimension of the first evolution level gives *d* = 4.1 with an undefined error, due to its divergence at *r* = 1. This implies that this fractal generator cannot be sustained in subsequent evolution steps, as it will overlap itself in space. As expected, the fractal dimension for the second level has a much lower value of *d* = 0.9 ± 0.1. The onset of fractal formation along the initial satellite chain is determined by the difference between $N_1^{\mathrm{c}}$ and *N*_1_ (shown in Fig. 3), as well as the sizes of secondary satellites relative to potential secondary cores. For example, if the $N_1 = N_1^{\mathrm{c}}$, then a fractal chain cannot form on the primary orbit and the fractal onset must happen further along primary chain. However, if the primary chain terminates at the primary orbit, then a splitting of the chain can occur (e.g., Fig. 7).

In general, the Hausdorff dimension of a fractal is always greater than its topological dimension^36^, with lines producing fractals with *d* > 1 and surfaces producing fractals with *d* > 2. In this problem, the fractals are formed by the self-assembly of smooth spheres that are separated by hyperbolic defects. This means that unlike the Apollonian sphere packing, in which their surfaces touch and fill all available space, there is no roughness associated with any of the surfaces of these structures. Instead, the concept of fractals manifests itself in the form of the tree-like structures of broken up components, such that *d* > 0 (cf. Cantor dust^37^ in 3D has *d* = log 8/log 3 ≈ 1.89). For a given evolution level, *d* describes the statistical distribution of inclusion sizes, as it represents the logarithmic ratio between the number of satellites and their sizes in relation to their core. We also observe that equal inclusion sizes have the tendency to form linear chains and a size jump is required to induce a fractal split. The only exception to this occurs at the primary core, where the split is symmetry driven. This means that all the relevant size distributions that are necessary to form fractal structures are described by the collection of fractal dimensions across the evolution levels in each nematic droplet.

Most of the first evolution structures from Fig. 3 have fractal dimensions in the *d* ∈ (1, 2) range, with a few exceptions close to *r* = 1. Since random motion is introduced during the creation of high *r* systems, fractal structures are also likely to form, which reduces the potential number of primary orbit satellites, as the branches extend laterally and push each other away. After each evolution step, both *N* and *r* typically increase, giving secondary evolution fractal dimensions in the *d* ∈ (0.75, 1.25) range. Due to the limits of optical resolution, fractal structures with higher orders of evolution become increasingly difficult to identify. The presence of further iterations was observed but were not individually distinguishable for accurate measurements. Additionally, the concept of a director field loses its meaning and becomes undefined over length scales comparable to the molecular scale. This imposes a lower limit onto the sizes of satellites and the number of possible fractal evolution steps. Beyond this point, liquid crystals cannot support topologically stabilized structures. On the other hand, the effects of boundary conditions on liquid crystals begin to lose coherence over distances larger than a few hounded μm. In this case, the fractal structures will still form locally but the directional symmetry will become increasingly less prominent with greater structure size. Figure 8b shows a nematic droplet with a 450 μm radius with an extremely complex fractal structure consisting of a large cascade of incoherent evolutionary steps.

## Discussion

In conclusion, we have investigated the properties of spontaneous self-assembly of geometric structures formed from water inclusion with normal boundary conditions inside radial nematic liquid crystal droplets. Due to the vector-like behavior of nematic liquid crystals, all disturbances in the director field are governed by topology rules. We created permanent disturbances by adding small water droplets with normal boundary conditions to the nematic droplet domains, which resulted in the formation of stabilizing hyperbolic defects in such  emulsions. Our results indicate that in geometrically unbiased nematic droplets, radial inclusions spontaneously form 3D structures with symmetry properties matching those described by the solutions to the classical Thomson problem. Using numerical simulations of the director field, we have shown that the ratio between the core and the satellite inclusions plays a key role in the resulting shapes of these self-assembled colloidal structures. We also provide a simple model to describe the maximal capacity of satellites around the core as a function of their size ratios and the elastic constants of the nematic liquid crystal. As expected, it suggests that as the ratio between the splay and the bend elastic constants gets bigger, a core can accommodate more satellites in its primary orbit. Similarly, a larger core can provide more room for director deformation and therefore, a higher number of satellites. The most common shape found experimentally consisted of a core with four dipolar satellite chains extending radially away from it with tetrahedral symmetry. The shape was then altered by physical agitation of the samples near the nematic to isotropic phase transition and deformation of spherical droplets to 2D disks to obtain single linear chains and triangular structures. Following this, we found that in systems with large distributions of satellite sizes, the colloids self-assembled into fractal structures. The number of symmetry evolutions depended on the distributions of satellite sizes. Systems with large distributions were able to achieve several evolutionary steps, surpassing the resolution of optical microscopy. Due to the length scales of director fields, the structures formed in nematic liquid crystals have a finite number of possible fractal evolution steps in the formation process of fractal colloids. This can be used for designing a variety of photonic structures with different complexity levels. For example, microfluidics can be used to create a core that accommodate a specific structure consisting of fluorescent intrusions or gold nano-particles.

## Methods

### Experimental

Room temperature nematic E7 liquid crystal mixture (from Synthon) and deionized water (containing 0.5 mass % of hexadecyltrimethylammonium bromide (CTAB) surfactant) were used to create fractal structures inside liquid crystal droplets. CTAB provides radial alignment and stabilizes the emulsions. Polydimethylsiloxane was used to create the double emulsion microfluidics devices in accordance to the methods described in ref. ^38^. Double emulsions of water in liquid crystal in water were achieved using a combination of three Harvard Apparatus PHD ULTRA syringe pumps and controlled shaking. Samples were extracted onto glass slides and covered with glass covering slips and studied using a Leica 2700 cross polarizing microscope. Optical microscopy photographs were taken using a Nikon D7100 camera. Counting the number of primary satellites was performed by drying the outer water phase, which caused the nematic droplets to roll and rotate the structures inside them. Confocal microscopy was performed using a Zeiss Elyra PS1 microscope with an alpha Plan-Apochromat ×100 oil immersion objective and captured using an Andor EMCCD detector.

### Numerical

Simulations of nematic director fields were performed using commercial finite element analysis software (COMSOL 5.3a) through a minimization of the Frank free energy^7^

4 $$E = \frac{1}{2}{\oint}_V \left[ {K_1(\nabla \cdot {\bf{n}})^2 + K_2({\bf{n}} \cdot \nabla \times {\bf{n}})^2 + K_3\left| {{\bf{n}} \times (\nabla \times {\bf{n}})} \right|^2} \right]{\kern 1pt} dV,$$

with the following values of Frank elastic constants *K*_1_ = 10.8 pN, *K*_2_ = 6.5 pN and *K*_3_ = 17.5 pN. The domain radius was set to be 30 μm, satellite size was fixed to 1 μm and *r* (ratio of the core to satellite radii) was varied from 1 to 10 in 0.1 step increments.

## Data Availability

The data that support the findings of this study are available in Research Data Leeds Repository with the identifier 10.5518/469 (ref. ^39^).
